# Early “Rootprints” of Plant Terrestrialization: Selaginella Root Development Sheds Light on Root Evolution in Vascular Plants

**DOI:** 10.3389/fpls.2021.735514

**Published:** 2021-09-04

**Authors:** Tao Fang, Hans Motte, Boris Parizot, Tom Beeckman

**Affiliations:** ^1^Department of Plant Biotechnology and Bioinformatics, Ghent University, Ghent, Belgium; ^2^VIB Center for Plant Systems Biology, Ghent, Belgium

**Keywords:** root branching, *Selaginella moellendorffii*, evolution, lycophyte, root meristem

## Abstract

Roots provide multiple key functions for plants, including anchorage and capturing of water and nutrients. Evolutionarily, roots represent a crucial innovation that enabled plants to migrate from aquatic to terrestrial environment and to grow in height. Based on fossil evidence, roots evolved at least twice independently, once in the lycophyte clade and once in the euphyllophyte (ferns and seed plants) clade. In lycophytes, roots originated in a stepwise manner. Despite their pivotal position in root evolution, it remains unclear how root development is controlled in lycophytes. Getting more insight into lycophyte root development might shed light on how genetic players controlling the root meristem and root developmental processes have evolved. Unfortunately, genetic studies in lycophytes are lagging behind, lacking advanced biotechnological tools, partially caused by the limited economic value of this clade. The technology of RNA sequencing (RNA-seq) at least enabled transcriptome studies, which could enhance the understanding or discovery of genes involved in the root development of this sister group of euphyllophytes. Here, we provide an overview of the current knowledge on root evolution followed by a survey of root developmental events and how these are genetically and hormonally controlled, starting from insights obtained in the model seed plant Arabidopsis and where possible making a comparison with lycophyte root development. Second, we suggest possible key genetic regulators in root development of lycophytes mainly based on their expression profiles in *Selaginella moellendorffii* and phylogenetics. Finally, we point out challenges and possible future directions for research on root evolution.

## Introduction

Whereas filamentous rhizoids fulfilled the “rooting” function of the first land plants (Jones and Dolan, 2012), true roots with a fully integrated vascular system developed in Early Devonian times and provided a much better ability to anchor large plants and absorb water and nutrients. Therefore, roots were an important innovation for successful colonization of land.

**Figure 1:**
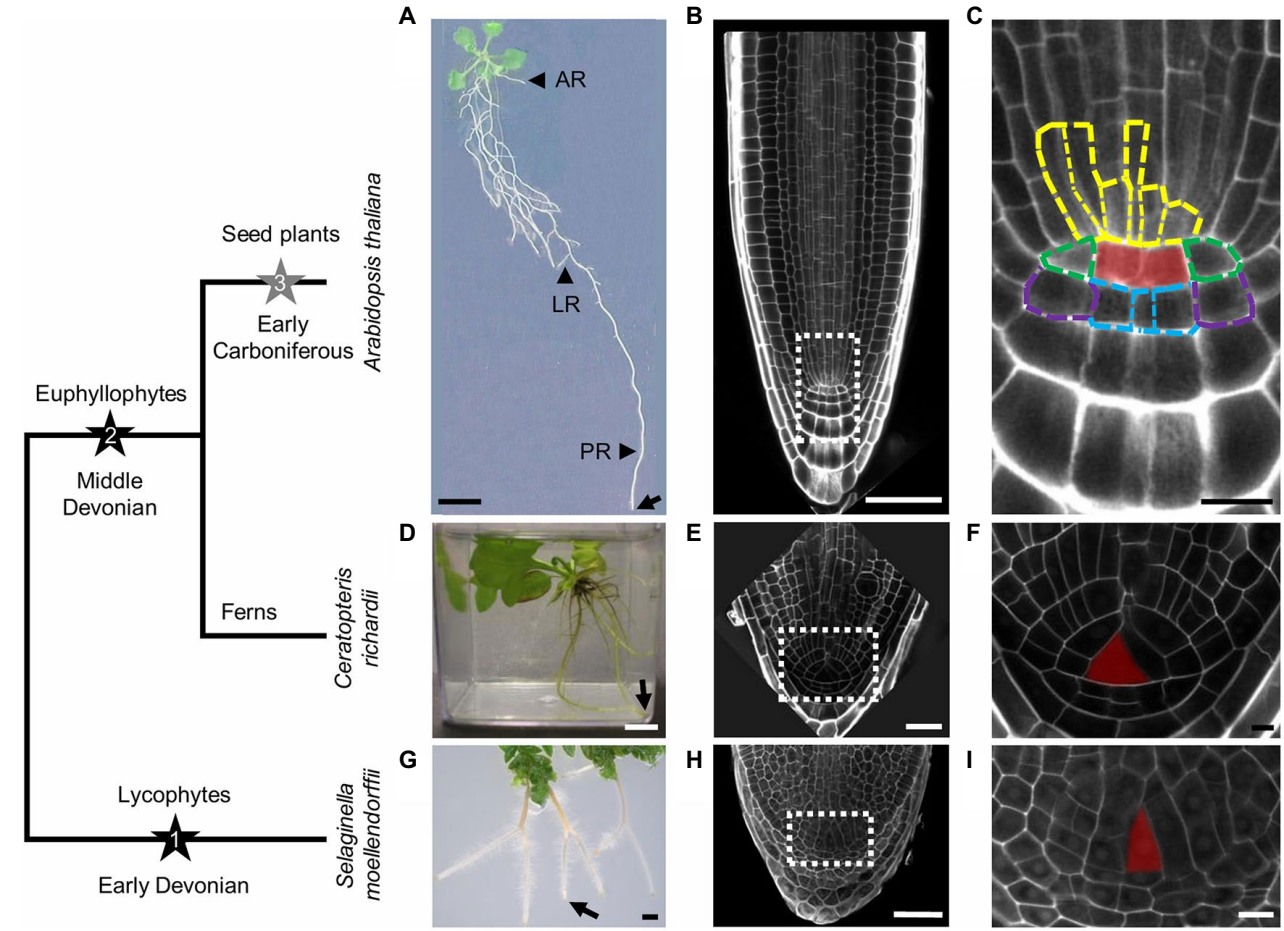
Simplified cladogram with images of rooting system and root meristem of the major groups of vascular plants. Black stars indicate the first two (1 and 2) valid independent root origins. It is currently unclear whether root evolution in seed plants can be seen as the third independent event (3), indicated by a gray star. **(A,D,G)** Rooting systems of *Arabidopsis thaliana* **(A)**, *Ceratopteris richardii* **(D)**, and *Selaginella moellendorffii* **(G)**. **(A)** In *A. thaliana*, adventitious roots (AR) and lateral roots (LR) arise from hypocotyl and primary root (PR), respectively. **(B,E,H)** Root meristems of *A. thaliana* **(B)**, *C. richardii* **(E)**, and *S. moellendorffii* **(H)**, which are magnifications of the root tips indicated by the arrows in **(A,D,G)**. **(C,F,I)** are magnifications as indicated by the dashed rectangles in **(B,E,H)**. **(C)** The quiescent center (red overlay) regulates or organizes the surrounding stem cells (initials) indicated by dashed outlines in different colors: vasculature initials (yellow), columella initials (blue), lateral root cap/epidermis initials (purple), and endodermis/cortex initials (green). In *C. richardii* **(F)** and *S. moellendorffii* **(I)**, the central apical cell or initial cell (red overlay) presumably organizes itself and is the sole root stem cell. Scale bars: 1cm **(A,D,G)**, 50μm **(B,E,H)**, and 10μm **(C,F,I)**.

In (extant) seed plants, a typical root system is composed of an embryonic primary root and postembryonic adventitious and lateral roots (Motte and Beeckman, 2019; Motte et al., 2020; Figure 1A). Crucial for their continuous growth is the development and maintenance of a root meristem, a tissue consisting of continuously dividing cells representing a source of cells to build the tissues of the main root. In seed plants, the root apical meristem (RAM) of the primary root is formed during embryo development while lateral root (LR) meristems are formed *de novo* in existing root tissues (Trinh et al., 2018). Both the development and maintenance of these meristems are controlled by a complex signaling network, including hormones, especially auxins, and transcription factors (TFs; Motte et al., 2019).

In extant non-seed vascular plants, different root systems can be found. In leptosporangiate ferns (e.g., *Ceratopteris richardii*), roots are shoot-borne and can form LRs, but in a rigid pattern (Hou et al., 2004; Hou and Blancaflor, 2018). In lycophytes, the first lineage where roots arose, and including the emerging model organism *Selaginella moellendorffii* (Chang et al., 2016), the embryonic root is short-lived, and the majority of the rooting system, consisting of root-bearing rhizophores and roots, is formed postembryonically (Mello et al., 2019). The rhizophore in Selaginella is a unique kind of root-bearing (from which roots develop) organ, a positive gravitropic leafless cylinder without typical root traits such as a root cap (RC) and root hairs (Nageli and Leitgeb, 1868; Mello et al., 2019). The transition from the rhizophore to the root is hallmarked by the appearance of these root traits (Lu and Jernstedt, 1996; Dolzblasz et al., 2018). Roots in Selaginella do not branch laterally, but at the tip (termed dichotomous root branching; Fang et al., 2019; Motte and Beeckman, 2019; Motte et al., 2020). Interestingly, fossil evidence reveals that there have been multiple origins for both the lateral and dichotomous branching patterns in root evolution (Hetherington et al., 2020), while dichotomous root branching seems to be conserved throughout lycophyte evolution (Hetherington and Dolan, 2017).

Intriguingly, the transition from rootless plants to the first root-bearing organisms did not require extra gene families, which suggests that the exploitation of existing genetic programs was sufficient for the generation of roots (Ferrari et al., 2020). Consistently, the number of TF families increased before but not during plant terrestrialization (Catarino et al., 2016). Although expansions of gene families is considered to underpin the evolution of gene function and biological innovations (Panchy et al., 2016), genomic analyses revealed that only limited expansions occurred at the divergence between the lycophyte and euphyllophyte clades (One Thousand Plant Transcriptomes Initiative et al., 2019; Wong et al., 2020). Thus, it seems that early root evolution might have adopted the functional co-option (new use of existing traits) of genes that duplicated in a large scale before emergence of vascular plants. Further on, root evolution has to be considered as an ongoing selective process instead of a sudden appearance, which is supported by anatomical (Fujinami et al., 2017) and fossil (Hetherington and Dolan, 2018b) evidence showing that roots gradually evolved multiple times to acquire traits in a stepwise manner within the lycophyte lineage. Moreover, paleobotanical evidence indicates that roots evolved at least twice, independently once in lycophytes and once in euphyllophytes (Raven and Edwards, 2001; Friedman et al., 2004; Figure 1). Nevertheless, gene expression programs seem to be conserved between these two lineages, suggesting the existence of an ancient root developmental program from the common ancestor of the vascular plants, or parallel recruitment of largely the same program to enable root development (Huang and Schiefelbein, 2015).

In this review, we first provide an overview of the current view on root evolution followed by an overview of the development, morphology, and anatomy of lycophyte roots (focusing on Selaginella). Furthermore, we survey the importance of auxins in root development of mainly Selaginella and speculate on the possible role of TFs for which evidence could be found in the conservation of their sequences and reported gene expression data in *S. moellendorffii*.

## Root Evolution

Land colonization by plants happened around 470 million years ago and is a milestone in plant evolution. It is generally believed that a bryophyte-like common ancestor of vascular plants developed rhizoids for rooting from the bottom surface of axes over 400 million years ago. Supportive for this, the extinct vascular lineage origin-spanning species, such as *Aglaophyton majus* and *Rhynia gwynne-vaughanii*, also developed bryophyte-like rhizoid-based rooting systems. Similar rooting systems were still preserved in the extinct early Devonian lycophytes, e.g., *Nothia aphylla*, which also lacked specialized rooting axes, i.e., sporophytic terminal axial organs performing rooting functions (Hetherington and Dolan, 2018a, 2019; Hetherington, 2019).

Roots evolved in a stepwise manner during lycophyte evolution. For example, a specialized rooting axe, with a continuous epidermal surface rather than a RC, was found in the extinct lycophyte *Asteroxylon mackiei* (Hetherington and Dolan, 2018b). This rooting organ deviates from the currently known roots of extant vascular plants that have a RC surrounding the RAM and indicates that roots did not appear from the start in their present form. Indeed, similarities with modern roots could be identified in less old fossilized lycophyte root meristems, dating back to over 300 million years ago, which showed a generally similar cellular organization with extant lycophyte root meristems (Hetherington et al., 2016).

Phylogenetic analyses considering fossil taxa demonstrated that roots evolved at least twice in vascular plants (Friedman et al., 2004; Hetherington and Dolan, 2018b, 2019), once in the lycophytes and once in the euphyllophytes, a sister clade of the lycophytes within the vascular plants. Euphyllophyte roots are anatomically similar to lycophyte roots, where an apical meristem provides cells for a multilayered main root with a central vasculature and typical root traits such as the RC and root hairs (Bierhorst, 1971). A characteristic generally interpreted as a sign of the dual origin is the different root branching strategy that is found in the extant vascular plants: (endogenous) LR branching in euphyllophytes and dichotomous (isotomous) root branching in lycophytes (Motte and Beeckman, 2019; Motte et al., 2020). However, recent paleobotanical evidence showed a different trajectory of euphyllophyte root evolution: (1) Dichotomous root branching was common in many early euphyllophyte groups during Devonian and Carboniferous periods; (2) LR branching evolved multiple times in at least three main euphyllophyte lineages independently: possibly first in the lignophytes (seed plants and progymnosperms, an extinct paraphyletic assemblage from which the seed plants evolved, including Archaeopteridales and Aneurophytales), second in Equisetopsida and third in ferns, including Marattiales and Leptosporangiate ferns (Hetherington et al., 2020). In contrast to the evolution of euphyllophyte root branching, root dichotomy seems to be conserved throughout lycophyte evolution (Hetherington and Dolan, 2017; Hetherington et al., 2020).

The living lycophytes consist of the orders Lycopodiales, Selaginellales, and Isoetales (PPG I, 2016). A first Selaginella genome was sequenced in *S. moellendorffii* (Banks et al., 2011) giving rise to multiple transcriptomic studies with root samples (Motte et al., 2020). Moreover, transient transfection of *S. moellendorffii* root protoplasts was used to test functioning of transcriptional responses (Mello et al., 2019). Thus, newly valuable omics resources and an expanded molecular toolbox advocate this species as an emerging representative in lycophyte (root) research. However, though genomes of several other Selaginella species have been sequenced (Ge et al., 2016; VanBuren et al., 2018; Xu et al., 2018), genomic resources of the other orders, i.e., Lycopodiales and Isoetales, still remain limited (Motte et al., 2020). In addition, the current molecular toolbox still needs to be much expanded. One of the greatest challenges in lycophyte research is to establish a (stable) transformation system, which would allow decent investigations into gene function using transgenics.

## RAM Organization

The RAM is crucial for plant roots as it forms a growing tip that supplies the root with new cells. To ensure this, it harbors one or more initials or stem cells, which do not differentiate but keep dividing to produce different cell types and to replenish the stem cell pool in the root. In Arabidopsis, the RAM contains a region of mitotically almost inactive cells, the quiescent center (QC), which is surrounded by different stem cells, including the initials for the vasculature, columella, lateral root cap/epidermis, and endodermis/cortex (Dolan et al., 1993; Motte et al., 2019; Figures 1B,C). The QC and the stem cells compose the root stem cell niche (SCN), in which the QC is important to maintain the identity of the stem cells (van den Berg et al., 1997; Sabatini et al., 2003).

Unlike Arabidopsis RAMs, the Selaginella RAM does not possess a QC but presumably only one stem cell called the initial cell (IC; Figures 1H,I). However, it is unknown how the identity of this initial cell is determined. The IC is presumably tetrahedral and probably cuts off daughter cells from four sides as the source of cells for the whole root (Imaichi and Kato, 1989; Figure 1I). Interestingly, the Selaginella RAM organization is quite similar to the organization of the fern RAM (Figures 1E,F). In some leptosporangiate ferns, the IC is also tetrahedral and divides a fixed number of times in a cyclic order at the three proximal sides, producing as such a fixed number of merophytes (packets of cells which are clonally related), which are stacked to form a root (Gunning et al., 1978; Hou and Blancaflor, 2018; Figure 1F). For instance, in the root apex of the fern *Azolla pinnata*, the IC divides 43 times to produce 12 successive merophytes, representing determinate root growth (Piekarska-Stachowiak and Nakielski, 2013). In addition, RC cells are produced from the IC distal face (Hou and Hill, 2004). A similar easy traceable cell division pattern is not obvious in Selaginella, and it is currently not entirely clear how a root in this plant is constructed.

Intriguingly, some other lycophytes, including *Lycopodium clavatum* and *Lycopodium diphasiastrum*, possess roots with a QC-like region, which contain cells with a slightly higher frequency of mitotic cell division than QC cells in the Arabidopsis root (Fujinami et al., 2017). In contrast, the lycophytes *Lycopodium obscurum* and Isoetaceae have no QC or QC-like region, but tiers of ICs from which different cell layers are derived (Yi and Kato, 2001; Fujinami et al., 2017). Such anatomic disparity of RAM organization in the extant lycophytes supports the idea that roots even evolved multiple times within this lineage.

## Root Branching

**Figure 2:**
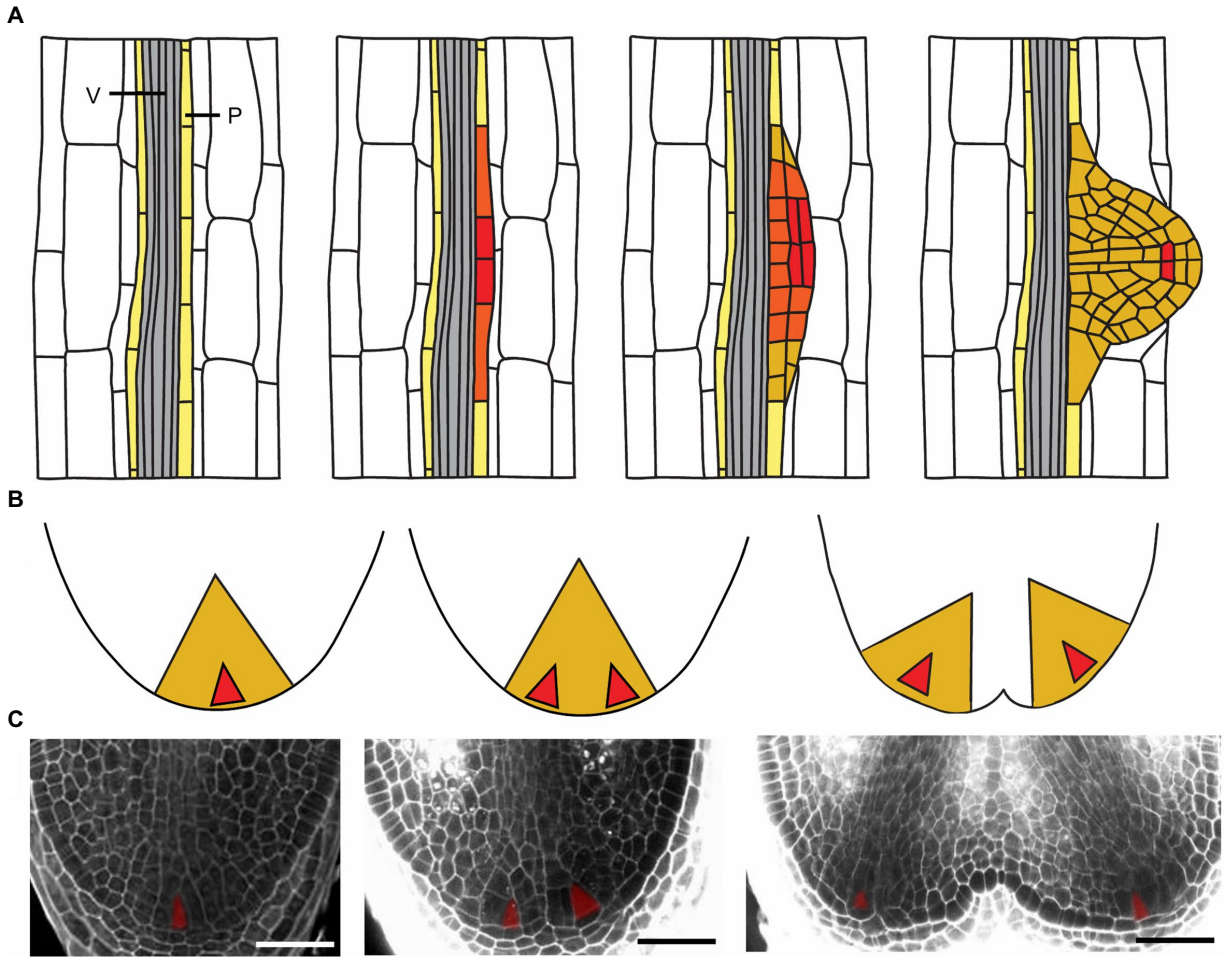
Root branching processes in Arabidopsis and Selaginella. **(A)** Scheme of LR development in Arabidopsis. An LR initiates in the xylem-pole pericycle cells (P, light yellow). After a series of anticlinal and periclinal cell divisions, an LR primordium is developed. Gray indicates vascular tissue (V). **(B,C)** Scheme and images of dichotomous root branching in *S. moellendorffii*. Shortly after the meristem starts to bifurcate, two young root primordia are formed with apical cells installed. The meristems further develop into more mature primordia. The color gradient, red–orange–dark yellow, respectively, indicates high–medium–low levels of stem cell-ness in all the root meristems. Scale bars: 50μm.

One of the advantages of seed plants during the colonization of land is their LR branching pattern, which is plastic and adaptable toward different conditions (Motte and Beeckman, 2019). In Arabidopsis, LR formation is well studied spatially and chronologically (Banda et al., 2019). LR formation in this species is initiated by nuclear migration and asymmetric divisions of two adjacent pericycle founder cells (Malamy and Benfey, 1997; Casimiro et al., 2001; Goh et al., 2012a); after initiation and a series of anticlinal and periclinal cell divisions, a new LR primordium is gradually formed, and a new SCN installed (Goh et al., 2016; Torres-Martinez et al., 2019; Figure 2A).

Lycophyte roots do not branch laterally like seed plants (Fang et al., 2019), possibly due to the lack of pluripotency of the pericycle cells. Instead, lycophyte roots branch dichotomously, and hence, two branches are formed at the root tip after bifurcation of the root meristem (Troll, 1937; Hetherington and Dolan, 2017; Fang et al., 2019). In Selaginella, formation of two ICs results in two new young root primordia (Otreba and Gola, 2011; Figures 2B,C). The primordia develop with continuity of vascular tissues and procambium preserved in both apices (Figure 2C). Until this phase, the branching is still well hidden inside the parent root tip (Otreba and Gola, 2011). After emergence from the parent root tip, the two new apices do not branch immediately and harbor only one IC in each RAM. Each RAM develops and will bifurcate again, a process that can be repeated several times as the root grows.

It is currently unknown which events are taking place to prepare Selaginella roots for branching. In addition, how two new ICs appear is still not clear: They are considered to emerge either after inactivation of the original IC (Imaichi and Kato, 1989; Otreba and Gola, 2011), or alternatively, a second IC might be derived from the original IC (Barlow and Lück, 2004). Investigation into the early branching events is required to answer this fundamental question. To our knowledge, only two papers described RAM bifurcation initiation in other lycophytes. In Isoetes, prior to branching, the apical meristem broadens and through a specific cell division pattern two rows of small and narrow non-meristematic cells are produced in the center of the meristem separating two groups of initials (Yi and Kato, 2001). In Lycopodium, a representative of the lycophytes having roots with a QC-like region as mentioned higher, dichotomous branching occurs by the appearance of actively dividing cells in the quiescent tissue. As a result, the parental meristem divides into two daughter meristems (Fujinami et al., 2021). The different bifurcation mechanisms within the lycophytes are probably reflecting the different RAM organization resulting from the gradual evolution of roots within this lineage.

## Auxin Control

Hormones play major roles in the control of root development and especially auxins are essential for RAM maintenance and LR formation, which is well-documented for Arabidopsis. To our knowledge, lycophyte root responses toward hormones have only been studied in Selaginellaceae, and mainly toward auxins.

Auxins, early characterized as “root forming hormones of plants” (Went, 1929), have long been known to regulate the development and maintenance of root meristems in plants. In particular auxin transport and, as a result, auxin gradients with increasing level toward the root tip is of utmost importance for this control. Arabidopsis root tips show an “inverted fountain” of auxin movement: auxin flows from the transition zone between meristem and elongation zone in a rootward direction and is then inverted in the RC through the epidermis and flows back to the transition zone. Both AUXIN1/LIKE-AUX1 (AUX/LAX) auxin influx carriers and PIN-FORMED (PIN) efflux carriers are important in this process (Trewavas, 1986; Gaillochet and Lohmann, 2015), which results in an auxin gradient with a maximum at the QC. This is crucial for the positioning of the QC and the surrounding stem cells (Sabatini et al., 1999; Shimotohno and Scheres, 2019). Similarly, auxin maxima and auxin signaling are crucial at different steps during LR formation (Cavallari et al., 2021).

Auxin biosynthesis, signaling, transport, and conjugation all predated evolution of vascular plants (Bowman et al., 2021). However, it is currently unknown whether auxin is also involved in the IC maintenance of the lycophyte RAM. In any case, auxin is, just as in other plants, rootwardly transported in Selaginella roots (Wochok and Sussex, 1974). Disturbance of this transport affects both root growth and meristem organization, whereas increase of auxin levels affects root growth, advocating for a role of an auxin gradient in the root meristem organization (Fang et al., 2019). Supportive for this, key components of auxin transport, e.g., AUX/LAXs and PINs, have also been identified in *S. moellendorffii* (Banks et al., 2011).

Moreover, exogenously applied auxins promote initiation of roots in Selaginella, whereas inhibitors of auxin transport prevent this initiation. The auxin indole-3-acetic acid (IAA; Williams, 1937) and auxin precursor indole-3-butyric acid (IBA; Webster, 1969) are even able to change the shoot fate to root fate of the angle meristem, located in the angles of shoot branches in many Selaginella species and giving rise to new shoots or rhizophores (Jernstedt et al., 1992; Banks, 2009). Likewise, the synthetic auxin 2,4-dichlorophenoxyacetic acid (2,4-D) promotes the root fate in the dorsal angle meristems (Mello et al., 2019). In contrast, an auxin transport inhibitor, 2,3,5-triiodobenzoic acid (TIBA), changes the angle meristems toward development of leafy shoots (Wochok and Sussex, 1975; Mello et al., 2019).

Other examples in Selaginella support a possible role of auxin in the root meristem development. Root-to-shoot conversion can be suppressed by the use of 1-naphthaleneacetic acid (NAA) in *Selaginella willdenowii* (Wochok and Sussex, 1976), and IBA can be used to initiate root cultures in *Selaginella microphylla* (Jha et al., 2013). On the other hand, IBA might also induce root to shoot conversion, indicating that not only auxin as such, but also a controlled balance of auxin levels, gradients, or signaling is possibly required during RAM establishment.

Furthermore, auxins also affect the dichotomous root branching in Selaginella. Different auxins promote proliferation activity in root tips, whereas high concentrations of the polar auxin transporter inhibitor, naphthylphthalamic acid (NPA), stop growth and branching (Fang et al., 2019), or result in callus-like tissue at the root tip (Sanders and Langdale, 2013). It is important to note though that auxins do not directly induce root branching in *S. moellendorffii*, suggesting that the branching initiation itself depends on an auxin-independent process (Fang et al., 2019).

## Comparative Genomics and Transcriptomics to Get Insights Into Lycophyte Root Development

Most of our current knowledge about genetic players in development of the primary root and LR is obtained from Arabidopsis research, which revealed a core set of TFs at a cellular level (recently reviewed by Motte et al., 2019 and Shimotohno and Scheres, 2019). Yet mechanisms controlling RAM activities remain elusive in lycophytes, and data that could highlight possible players are mainly restricted to comparative genomics or gene expression data.

Recently, multiple tools to analyze gene expression data from *S. moellendorffii* and to perform comparative studies with other plant species have become available such as the recently designed Co-expression Network Toolkit (CoNekT; Proost and Mutwil, 2018), in which Ferrari et al. (2020) integrated different publicly available *S. moellendorffii* RNA-seq datasets. Additionally, Ferrari et al. (2020) designed the Selaginella eFP Browser, which provides color-coding pictographic representations for the gene expression level in different organs or tissues (Winter et al., 2007). As auxins seem to play a role in RAM establishment and maintenance of vascular plants, we surveyed the representative gene families that are, respectively, crucial in auxin biosynthesis, signaling, transport, and metabolism as well as important transcriptional regulators and highlight their possible role in Selaginella root development mainly based on phylogenetic and transcriptomic studies.

### Auxin Biosynthesis

TRYPTOPHAN AMINOTRANSFERASE (TAA) and YUCCA FLAVIN-DEPENDENT MONOOXYGENASE (YUC/YUCCA) gene families play a crucial role in auxin biosynthesis in plants (Mashiguchi et al., 2011). Members regulate plant root development, as reviewed by Olatunji et al. (2017). TAA proteins likely originated during chlorophyte evolution (Mutte, 2020), whereas the origin of YUCs is unclear. An ancient divergence into the clades YUC and sYUC occurred during charophyte evolution, while the sYUC clade disappeared in Arabidopsis (Mutte, 2020). Furthermore, *YUC* genes belong to a deeply conserved auxin-dependent gene set with similar regulation patterns shared by all land plants (Mutte et al., 2018).

In Arabidopsis, the highest auxin synthesis rate of the root is detected in the RAM (Ljung et al., 2005). Specifically, auxin is locally produced in the QC, which is required for RAM maintenance (Casanova-Saez and Voss, 2019). Mutations in the *TAA* genes *TAA1* and *TAA1-RELATED2* (*TAR2*) result in reduced root meristematic activity (Stepanova et al., 2008), whereas most *YUC* mutants do not even form a root meristem (Cheng et al., 2007), demonstrating their importance in RAM maintenance and establishment. Additionally, some *YUC* genes are also expressed at early stages during LR formation (Hentrich et al., 2013; Cai et al., 2014; Tang et al., 2017), suggesting a possible role during LR development as well.

*Selaginella moellendorffii* has one *TAA* homologue, which does not have specific or high expression in the root or RAM (Ferrari et al., 2020), suggesting a possibly limited role in lycophyte root development. Interestingly, an auxin biosynthesis inhibitor that competitively inhibits YUC enzymes reduces root growth in *S. moellendorffii* (Kaneko et al., 2020). Additionally, transcripts of two *sYUC* genes accumulate substantially in the *S. moellendorffii* root and one of them is also highly expressed in the RAM (Table 1). On the contrary, transcripts of the three homologues from the YUC clade only have low abundance. Thus, in particular, auxin biosynthesis *via* sYUC homologues might be important in the establishment or maintenance of the root meristem of lycophytes.

**Table 1:** Specific and high root and root apical meristem (RAM) expression of the auxin-related gene family members in *S. moellendorffii*.

Gene family	Clade	Function	Gene ID	Arabidopsis homologue name	SRE	HRE	S RAM E	H RAM E
YUC	sYUC	Auxin biosynthesis	Smo113792	N/A		+		
YUC	sYUC	Auxin biosynthesis	Smo422043	N/A		+		+
TIR1/AFB	TIR1/AFB	Auxin receptor	Smo170974	TIR1, AFB1-5		+		
Aux/IAA	ncIAA	Auxin signaling	Smo415204	IAA33				+
Aux/IAA	ncIAA	Auxin signaling	Smo417391	IAA33				+
PIN	Lyco	Auxin transport	Smo119024	N/A	+			+

+: specific root/RAM expression [SRE / S RAM E, specific measure (SPM)>0.85]. SPM was calculated in the CoNekT (Proost and Mutwil, 2018; Ferrari et al., 2020) using the Tissue Specificity (root) or Condition Specificity (RAM) method. High root/RAM expression (HRE / H RAM E): shown red color based on absolute value in Selaginella eFP Browser (Winter et al., 2007; Ferrari et al., 2020). Published phylogenetic trees were prioritized for homologue inference, and OrthoFinder (v1.1.8 in CoNekT) was alternatively used to also identify robust homologues within the same orthogroup (Emms and Kelly, 2015, 2019). N/A, not available.

### Auxin Signaling

The core components of auxin signaling are TRANSPORT INHIBITOR RESPONSE 1/AUXIN SIGNALING F-BOX (TIR1/AFB) auxin receptors, AUXIN/INDOLE-3-ACETIC ACID (Aux/IAA) transcriptional repressors, and AUXIN RESPONSE FACTOR (ARF) TFs (Perrot-Rechenmann, 2014; Leyser, 2018). Auxin binds TIR1/AFB-Aux/IAA co-receptors, which leads to degradation of the Aux/IAAs and release of ARF TFs that regulate auxin responsive genes (Gray et al., 2001; Dharmasiri et al., 2005; Tan et al., 2007; Dos Santos Maraschin et al., 2009; Korasick et al., 2014; Israeli et al., 2020). Such a complete auxin response system is present in all land plants, but increased in complexity during evolution (Bowman et al., 2021). Phylogenetic analysis shows that the *Aux/IAA* gene family diverged into canonical and noncanonical *Aux/IAAs*. The latter do not bind to TIR1/AFB and cannot form a co-receptor. The ARF family split into class A, B. and C ARF subfamilies (Mutte et al., 2018), with class A ARFs being transcriptional activators, whereas the B or C classes are repressors.

In Arabidopsis, various Aux/IAA-ARF modules, involving canonical Aux/IAAs and all ARF classes, are involved in LR formation, embryonic RAM initiation, or RAM maintenance (Dello Ioio et al., 2008; Ding and Friml, 2010; Goh et al., 2012b; Palovaara et al., 2016; Promchuea et al., 2017; Du and Scheres, 2018). Interestingly, also the noncanonical IAA33 controls root stem cell identity *via* interaction with ARF10 and ARF16 (Lv et al., 2020), belonging to the class C. The orthologue of ARF16 also seems to be involved in RAM initiation in the conifer *Pinus pinaster* (de Vega-Bartol et al., 2013).

In *S. moellendorffii*, one *TIR1* homologue is highly expressed in the root and two homologues of *IAA33* show high expression in the RAM (Table 1), but none of the *ARF* homologues show specific or high expression in the root or RAM (Ferrari et al., 2020). Thus, it seems possible that noncanonical *IAAs* play a role in the meristem, whereas the role of *ARF* genes may be limited. Mello et al. (2019) further demonstrated that the transcriptional auxin responses function in *S. moellendorffii*, using auxin-treated root protoplasts transfected with a DR5 auxin response marker.

### Auxin Transport

Polar auxin transport is believed to be a key part of a molecular toolkit used by the early streptophytes toward a better adaptation to land conditions (Bennett et al., 2014; Bennett, 2015). PIN proteins that are auxin efflux transporters direct polar auxin transport to regulate development of the RAM and the LR meristem, which has been intensively studied in Arabidopsis (Motte et al., 2019). A duplication occurred within the lycophytes, producing two PIN subclades (Lyco PIN1 and Lyco PIN2), which are sister to all the euphyllophyte subclades: Eu1-3 (Bennett et al., 2014; Bennett, 2015). The protein sequences of the lycophyte and euphyllophyte clades are similar, but differences exist. For instance, PIN2 has a particular hydrophilic loop domain that originated during seed plant evolution and that is crucial to mediate fast gravitropic response of the root for good adaptation to dry land (Zhang et al., 2019).

In Arabidopsis, PIN1 and PIN3 play key roles in RAM establishment and LR initiation (Friml et al., 2003; Marhavy et al., 2013; Chen et al., 2015). In addition, expression of PIN proteins is induced by auxins in the root (Vieten et al., 2005). Intriguingly, in contrast to seed plants, the fern Azolla does not show an increased RAM size when treated with auxins, nor an induction of *PIN* expression (de Vries et al., 2016), which suggests a different mechanism in the control of meristem size compared to Arabidopsis.

In *S. moellendorffii*, representative PINs failed to replace the fast root gravitropism of *AtPIN2* (Zhang et al., 2019). Particularly PINV may play an important role in generation of the root meristem, as *PINV* is specifically expressed in the *S. moellendorffii* root and the transcripts accumulate at a high level in the RAM (Table 1). In the gametophyte-dominant bryophyte Physcomitrella, PINs also drive meristem function as auxin transport facilitators (Bennett et al., 2014). Intriguingly, a recent study utilized extensive cross-species functional complementation experiments with *PIN* genes from different streptophyte lineages, showing that the shoot/root development function, e.g., establishment of auxin maxima at the root tip, actually originated in land plants (Zhang et al., 2020).

### Auxin Metabolism

The GRETCHEN HAGEN3 (GH3) enzyme family conjugate compounds including auxin to amino acids, in order to control auxin homeostasis, which has an important role in plant development such as root growth (Casanova-Sáez et al., 2021). Phylogenetically, GH3s are classified into three groups: I–III (Chiu et al., 2018).

In Arabidopsis, a group II member GH3.17 is involved in the formation of auxin minima, which regulates RAM size (Di Mambro et al., 2017). In addition, the other group II genes regulate LR formation with a possible involvement in root pre-patterning by controlling levels of IBA-derived auxin in the RC (Xuan et al., 2015).

In *S. moellendorffii*, GH3s, especially the group II, play a predominant role in auxin homeostasis (Kaneko et al., 2020). However, the only homologue of Arabidopsis group II genes does not show specific or high expression in the root or RAM (Ferrari et al., 2020).

### Developmental Genes

Gene families, such as AINTEGUMENTA (ANT), GRAS [for GIBBERELLIC ACID INSENSITIVE (GAI), REPRESSOR OF GA1 (RGA), and SCARECROW (SCR)], and WUSCHEL (WUS)-LIKE HOMEOBOX (WOX), contain genes that play diverse roles in plant signaling and development. Some of these gene family members are important root stem cell regulators (Motte et al., 2020). In Arabidopsis, *PLETHORA* genes, which are ANT gene family members, control QC specification and stem cell activity, with a concentration gradient closely associated with auxin maxima (Aida et al., 2004). The GRAS member SHORTROOT (SHR) is expressed in the root vascular tissue and moves to the QC, initial of cortex and endodermis, as well as endodermis in Arabidopsis (Helariutta et al., 2000; Nakajima et al., 2001; Cui et al., 2007; Augstein and Carlsbecker, 2018). In these cells, another GRAS family member SCR forms a heterodimer with SHR (Hirano et al., 2017; Hakoshima, 2018), playing a key role in root stem cell control. In addition, SCARECROW-LIKE 23 (SCL23), which is encoded by the closest homologue of *SCR*, acts redundantly with SCR in the SCN (Long et al., 2015). The WOX family member *WOX5* is expressed in the QC and the WOX TF moves to the adjacent stem cells, preventing them from differentiation in the SCN (Sarkar et al., 2007; Kong et al., 2015). Moreover, *WOX13* is expressed in RAM stem cells, suggesting possible importance in root meristem formation (Deveaux et al., 2008).

The above-mentioned TFs also interact with each other during the regulation of meristem activity. For example, PLTs constrain the expression domain of WOX5 in the SCN, in which they maintain the QC and regulate the fate of columella stem cells (Burkart et al., 2019). SCR physically interacts with PLT, as well as TEOSINTE-BRANCHED1/CYCLOIDEA/PCF20 (TCP20), which induces *WOX5* expression to specify the SCN (Shimotohno et al., 2018). In turn, *WOX5* interacts with *SHR/SCR* and auxin pathways to maintain the SCN, preserving the QC identity (Sarkar et al., 2007).

These key regulators also function in the LR meristem formation: *PLT3*, *PLT5*, and *PLT7* are expressed early in the stage I primordium where the by them controlled asymmetric cell division occurs afterward to give rise to the stage II primordium. In addition, *PLT1*, *PLT2*, and *PLT4* are expressed later during LR outgrowth (Du and Scheres, 2017). SHR is crucial for LR development, including initiation and the control of asymmetric divisions of cortex/endodermis initials (Lucas et al., 2011). Besides, SHR activates *SCR* expression, which is the key for LR QC formation (Goh et al., 2016). Similarly as in the primary root, *WOX5* is during LR formation induced by a joint activity of PLTs, TCP20, and SCR (Shimotohno et al., 2018). Moreover, *WOX13* is not only expressed at the early stage of LR development, but also expressed during LR emergence (Deveaux et al., 2008). To investigate the possible significance in lycophyte root development, we next survey these gene families and highlight their possible roles in the lycophyte *S. moellendorffii*.

#### ANT

Based on a recently updated phylogenetic study, the ANT family can be divided into three clades: preANT, basalANT, and euANT; divergence of the ancestral preANT into two land plant-specific clades (basalANT and euANT) is hypothesized to be involved in plant terrestrialization (Dipp-Alvarez and Cruz-Ramirez, 2019). The most recently diverged euANT lineage, which has been intensively studied in Arabidopsis, includes members such as PLTs and ANT. Within the euANT lineage, two major sister clades can be found: one including AtANT and AINTEGUMENTA-like1 (AtAIL1), and the other including all the PLTs of Arabidopsis (Kim et al., 2005; Floyd and Bowman, 2007; Dipp-Alvarez and Cruz-Ramirez, 2019).

In *S. moellendorffii*, five genes were retrieved in this family: two in the euANT lineage and the other three in the basalANT lineage (Dipp-Alvarez and Cruz-Ramirez, 2019). All lycophyte euANT members fall within the ANT/AIL1 clade. Still, their motifs also overlap with the Arabidopsis PLT-specific motifs (Motte et al., 2020). One of the *S. moellendorffii* euANT homologues has a high expression in the RAM (Table 2), which may point to a possible role in the lycophyte RAM. Thus, it could be that the euANT TFs have conserved roles in RAMs of land plants.

**Table 2:** Specific and high root and RAM expression of the developmental genes in *S. moellendorffii*.

Gene family	Clade	Gene ID	Arabidopsis homologue name	SRE	HRE	S RAM E	H RAM E
ANT	euANT	Smo96572	ANT, AIL1				+
GRAS	SCR	Smo84762	SCR, SCL23		+		+
GRAS	SHR	Smo12696	SHR, SCL29/32	++			
GRAS	SHR	Smo53339	SHR, SCL29/32		+		
GRAS	SHR	Smo64241	SHR, SCL29/32		+		
GRAS	SHR	Smo90295	SHR, SCL29/32		+		
WOX	T1WOX	Smo4561	WOX10/13/14	+		+	+

+: specific root/RAM expression [SRE / S RAM E, specific measure (SPM)>0.85]; ++: exclusive expression (SPM=1). SPM calculated in the CoNekT (Proost and Mutwil, 2018; Ferrari et al., 2020) using the Tissue Specificity (root) or Condition Specificity (RAM) method. High root/RAM expression (HRE / H RAM E): shown red color based on absolute value in Selaginella eFP Browser (Winter et al., 2007; Ferrari et al., 2020). Published phylogenetic trees were prioritized for homologue inference, and OrthoFinder (v1.1.8 in CoNekT) was alternatively used to also identify robust homologues within the same orthogroup (Emms and Kelly, 2015, 2019). N/A, not available.

#### GRAS

GIBBERELLIC ACID INSENSITIVE (GAI), REPRESSOR OF GA1 (RGA), and SCARECROW (SCR) genes are believed to be incorporated into the common ancestor of Zygnematophyceae (the likely sister group to land plants) and land plants, *via* horizontal gene transfer from soil bacteria, to regulate processes from development to defense against various stresses during early land colonization. *GRAS* genes also expanded in the common ancestor, which is believed to be relevant for the evolution and radiation of land plants after divergence (Cheng et al., 2019). Ancient diversification of *GRAS* genes into different major clades occurred before divergence of the moss and vascular plants (Engstrom, 2011). Among the clades, *SHR* and *SCR* are representatives from the two clades of SHR and SCR, respectively (Bolle, 2004, 2016).

Interestingly, the *S. moellendorffii* genome contains, relatively to its genome size, more *GRAS* genes than Arabidopsis (Song et al., 2014). In *S. moellendorffii*, five genes were retrieved belonging to the SHR clade, and two genes for the SCR clade (Wang et al., 2016; Zhang et al., 2018). All but one Selaginella *SHR* gene have either high root expression, or have exclusive root expression (Table 2), suggesting a possible role of this clade in lycophyte root development. In addition, one homologue of *SCR* and *SCL23*, from the SCR clade, is highly expressed in the root and more specifically, in the RAM as well (Table 2). Thus, the expression of multiple *SHR* and *SCR* homologues is associated with the root meristem, and the SHR-SCR function might possibly be (partially) conserved in vascular plants.

#### WOX

WUSCHEL-LIKE HOMEOBOX can be divided into three superclades, which were recently termed Type 1 (T1WOX, the WOX10/13/14 clade), Type 2 (T2WOX, the WOX8/9 and WOX11/12 clades), and Type 3 (T3WOX, the WUS, WOX1/6, WOX2, WOX3, WOX4 and WOX5/7 clades) (Wu et al., 2019).

In the fern *C. richardii*, T2*WOX* genes, *WOXA* and *WOXB* are, respectively, expressed in the root mother cells and throughout the root meristem; the T3*WOX* member *WUSCHEL-LIKE* (*WUL*) is expressed in the root tips, whereas the T1*WOX* members *WOX13A* and *WOX13B* do not have specific root expression (Nardmann and Werr, 2012). In *S. moellendorffii*, eight *WOX* genes can be retrieved (Nardmann et al., 2009). Only one Selaginella *WOX* gene has specific and high expression in the RAM (Table 2), implying a possible role in the lycophyte root meristem. This *WOX* gene is a T1WOX member, lacking the canonical WUS-box, a conserved motif shared within the WUS subclade that is, at least in angiosperms, required for stem cell regulation and repressive transcriptional activities (Dolzblasz et al., 2016; Zhou et al., 2018). Thus, this T1WOX member, if having a function in stem cell specification, probably works *via* a different mechanism.

## Discussion and Conclusion

Fossil records of extinct lycophytes argue that true roots were absent in the ancient lycophyte trees and that a modified shoot system was co-opted to execute root functions (Kenrick, 2013). During early root evolution, lycophyte roots acquired root traits in a stepwise manner (Hetherington and Dolan, 2018b, 2019). Consistently, different extant lycophyte species have various types of RAM organization (Fujinami et al., 2017, 2020). Thus, it is conceivable that multiple root origins occurred during lycophyte evolution. Interestingly, branching of the root system in different patterns predated root evolution (Hao et al., 2010; Matsunaga and Tomescu, 2016; Rothwell and Tomescu, 2018), but the dichotomous branching pattern was preserved in the extant lycophyte roots. Insights in the dynamics of lycophyte RAM organization and initiation of root branching still await breakthroughs in molecular technologies and application of advanced imaging methods, e.g., live imaging.

How the evolution of plant growth hormones has been associated with root evolution is largely elusive. In the case of auxin, emergence of the core components mediating the response clearly predated lycophyte evolution (Bowman et al., 2021). In contrast to seed plants, auxins cannot induce root branching in non-seed vascular plants (Hou and Hill, 2004; Fang et al., 2019). To better understand hormonal pathways controlling development of lycophyte root meristems, the effect of more hormones, for example cytokinin, ethylene, and abscisic acid, should be experimentally tested and physiologically and genetically evaluated.

It is intriguing that lycophytes utilized the same set of probably only slightly expanded gene families for root evolution (One Thousand Plant Transcriptomes Initiative et al., 2019; Ferrari et al., 2020). Consistently, the majority of TF families evolved before land colonization of plants (Catarino et al., 2016). Thus, it is plausible that the rootless common ancestor of vascular plants co-opted the present genetic material for root evolution. Supportive for this, important developmental gene families, which are reviewed here, might play central roles in root meristem maintenance of lycophytes.

As gene families evolved and expanded (One Thousand Plant Transcriptomes Initiative et al., 2019; Wong et al., 2020), functional divergence will have occurred. Here, we mainly used *S. moellendorffii* expression data to predict the function, as stable transformation is currently unavailable in lycophyte research, obstructing functional investigation. We fully realize that the expression data alone do not legitimate to conclude on functional conservation or divergence of lycophyte genes. More experimental approaches, such as *in situ* hybridization, cross-species functional complementation and sequence domain analysis, may help to better understand root-function evolution of the gene families. We hope that our study is able to motivate the community to collect more such early “rootprints” of lycophytes, which would allow us to see clearer evolutionary trajectories of the root in vascular plants.

## Data Availability Statement

The original contributions presented in the study are included in the article, further inquiries can be directed to the corresponding author.

## Author Contributions

TF conceptualized the manuscript. TF, HM and TB wrote the manuscript. HM made the figures. All authors contributed to the article and approved the submitted version.

## Funding

This study was financially supported by the Fonds voor Wetenschappelijk Onderzoek – Vlaanderen (FWO)-projects G027313N and G028421N. TF was financially supported by China Scholarship Council (CSC) and Lotus Unlimited Project in the Erasmus Mundus program of the European Union.

## Conflict of Interest

The authors declare that the research was conducted in the absence of any commercial or financial relationships that could be construed as a potential conflict of interest.

## Publisher’s Note

All claims expressed in this article are solely those of the authors and do not necessarily represent those of their affiliated organizations, or those of the publisher, the editors and the reviewers. Any product that may be evaluated in this article, or claim that may be made by its manufacturer, is not guaranteed or endorsed by the publisher.
